# A Comparison of Mothers’ Quality of Life after Normal Vaginal, Cesarean, and Water Birth Deliveries

**Published:** 2015-07

**Authors:** Zahra Kavosi, Ali Keshtkaran, Fatemeh Setoodehzadeh, Maryam Kasraeian, Mohammad Khammarnia, Marzieh Eslahi

**Affiliations:** 1 Department of Health Services Management, School of Management and Medical Information, Shiraz University of Medical Sciences, Shiraz, Iran;; 2 Department of Obstetrics & Gynecology, Medical School, Shiraz University of Medical Sciences, Shiraz, Iran;; 3 Health Promotion Research Center, Zahedan University of Medical Sciences, Zahedan, Iran;; 4 Student Research Committee, Shiraz University of Medical Sciences, Shiraz, Iran

**Keywords:** Cesarean section, Natural childbirth, Pregnancy, Quality of life

## Abstract

**Background:**

Assessment of quality of life (QOL) is of paramount importance for improving postpartum QOL which will in turn enhance QOL of mothers, children, individuals, and the community. The present study aimed to evaluate and compare postpartum QOL after Cesarean Section (CS), Normal Vaginal Delivery (NVD), and water birth delivery.

**Methods:**

This descriptive analytical, cross-sectional study was conducted on postpartum women referred to urban health centers and two public hospitals in 2012-13 in Shiraz, Iran. Overall, 59 women with NVD, 39 with CS, and 39 with water birth, all at 2 months postpartum, were recruited into the study through multi-stage sampling. Postpartum QOL was measured using Short Form Health Survey (SF-36) which hadbeen adapted previously in Iran. Then, the data were analyzed using descriptive statistics and one-way analysis of variance (ANOVA) in SPSS, version 18.

**Results:**

The results showed that the NVD group had the highest mean score in physical health domains; the women with water birth had the highest mean score in mental health domains and total QOL. Regarding postpartum QOL the results of one-way ANOVA showed no statistically significant differences between the three modes of delivery.

**Conclusion:**

Women with water birth and NVD had the highest and second highest total QOL mean scores, respectively; women with NVD and water birth experienced better physical health. Thus, providing more information to pregnant women to encourage them to use NVD and water birth is suggested.

## Introduction

Delivery is a physiological process^1^ and is of paramount importance in health-care systems worldwide.^2^ It is also a critical life event for women. It is a multi-dimensional process involving physical, emotional, physiological, cultural, and mental aspects.^3^ Yet, labor pain is a major concern for pregnant women.^4^ It is also the primary cause of women’s avoiding Normal Vaginal Delivery (NVD) and tendency toward Cesarean Section (CS) as an alternative.^5^ Cesarean section is defined as abdominal and uterus incision in order to allow the baby, placenta, and extra embryonic membranes to be taken out.^6^ CS is a surgical intervention to prevent maternal or perinatal complications^7^ and must be limited to high -risk complicated pregnancies. World Health Organization (WHO) recommended the rate of CS be decreased to 15% in 2010. Despite WHO’s recommendation, the rate of CS has dramatically risen over the past decades.^8^ This rate was reported to vary from 7% to 22% in Finland, Sweden, and England.^4^ In Iran, investigations have shown that CS rate is far higher compared to European countries. According to 2005 publications, 47% of the deliveries in Iran were carried out by CS.^9^In recent years, pregnant women have been faced with many choices among new modes of delivery.^10^ Researchers have also become interested in developing non-pharmaceutical strategies for lowering labor pain. In general, medical teams and pregnant women approve of non-pharmaceutical pain-controlling strategies due to their comfort, possibility of more emotional support, and safety of both mother and her infant.^11^ Water birth is a non-pharmaceutical low-pain mode of delivery.^4^ This mode increases pain threshold and contributes to effective uterine contractions, facilitating the delivery process with no need for pharmaceutical interventions.^5^ Water birth was introduced in the late 1990s. Currently, it accounts for 38% of deliveries in New Zealand.^12^ In Iran, a framework for mother friendly hospitals was introduced in 2006. Developing various modes of painless or low pain normal delivery was incorporated into this framework as an objective.^5^ Despite more than 20 years of worldwide water birth in warm water pool, it is not wildly established in Iran’s hospitals and most pregnant women and healthcare providers are not familiar with this method.

Besides, assessment of quality of life (QOL) is important for Improving postpartum QOL and will enhance the QOL of mothers, children, individuals, and the community. WHO defines QOL as an individual’s perception of his/her life status. QOL includes physical, mental, and social aspects.^13^ In health promotion planning, it is crucially important to measure quality of life, which includes life status, socio-environmental factors, attitudes, interests, individuals’ goals, and social values.^14^ Studies have indicated that the postpartum period is a critical life occurrence for women, leading to changes in their health.^15^^,^^16^ Women’s experiencing it influences their physical health conditions and may affect their QL and future health.^7^ Some women suffer from postpartum depression, exhaustion, illness, insomnia, breast tenderness, physical pain, constipation, and sexual disorders which are largely related to delivery mode.^17^

Up to now, several studies have investigated postpartum QOL after NVD and CS.^16^^,^^18^ However, few studies have evaluated the impact of water birth, as a new mode of delivery, on postpartum QOL. Besides, contradictory results have been reported regarding the relationship between quality of life and mode of delivery.^7^^,^^19^ Enhancing mothers’ postpartum QOL guarantees and improves the QOL of children, family and society^7^ and it is associated with such factors as type of delivery and application of new delivery modes in health systems; therefore, the present study aimed to investigate the impact of mode of delivery on women’s quality of life after NVD, CS, and water birth. The study results can assist policy-makers and health system authorities to provide high quality services.

## Materials and Method

This descriptive-analytical, cross-sectional study was conducted on women who had had NVD, CS, and water birth and were at 2 months postpartum in two public hospitals and urban health centers from June 2012 to Feb 2013 in Shiraz, Iran. Based on the results of the previous studies^18^ and considering the significance level of 0.05, a 137-subject sample size was determined for the study. Using the following statistical formula, the sample size was determined at 59 women for vaginal delivery, 39 women for water birth and 39 women for cesarean section.

n= (z1-∝2+z1-β)2⁡(∝12+∝2/k2)(μ2-μ1 )2

µ_1_=21.66

µ_2_=23.38

α=0.05 

β=0.2 

k=1.5 

n2=1.5n1, n3

As most women in the city of Shiraz are covered by maternal and child health programs in public health centers, we selected women who had had vaginal delivery and cesarean section from these centers using a multi-stage sampling method. In the first stage, all Shiraz health centers were clustered into nine and from each cluster, one health center was randomly selected (simple random sampling). Then, from each health center women who had delivered in the previous two months or more and met the inclusion criteria were selected through purposive sampling. Only two public hospitals provide water birth services in Shiraz, and the limited number of all women who had had water birth in those two hospitals and met the inclusion criteria were enrolled in the study. The inclusion criteria of the study were being from Iran, aged between 20 and 32 years, being able to read and write, having undergone delivery in a public hospital and nulliparous women. The exclusion criteria were mother’s suffering from diagnosed psychological and medical problems, infant death or defect, and separation or divorce from one’s spouse. 

QOL was measured using the Short Form Health Survey (SF-36). SF-36 is a general instrument for measuring the quality of life. “It consists of 36 items classified into eight scales, namely Physical Functioning (PF), role limitation due to physical problems or Role Physical (RP), Bodily Pain (BP), General Health (GH), Vitality (VT), Social Functioning (SF), role limitation due to emotional problems or Role Emotional (RE), and Emotional well-being (EW)”.^20^ The score of each subscale could range from 0 to 100, with higher scores indicating better conditions. In fact, SF-36 evaluates physical health (based on PF, RP, BP, and GH scales) as well as mental health (based on VT, SF, RE, and EW).^7^ Consequently, the questionnaire presents a total QOL. It should be noted that BP and RP and RE have reverse scores SF-36 has been adapted in Iran. Investigation of internal consistency of the Persian version of SF-36 questionnaire showed minimum reliability standard coefficients ranging from 0.77 to 0.9. In addition, assessment of content validity showed satisfactory correlation coefficients (0.58-0.95) which were higher than the standard level; i.e., 0.4.^20^

The study was approved by the Ethics Committee of Shiraz University of Medical Sciences (SUMS), Shiraz, Iran. The participants gave their informed consent to participate, and were informed that participation in and withdrawal from this study were voluntary.

Minimum, maximum, and mean scores of the participants’ quality of life were calculated through descriptive statistics. Besides, one way analysis of variance (ANOVA) was used to determine the differences among the three delivery modes regarding the quality of life scores. We used SPSS, version 18 and the level of significance was set at 0.05. 

## Results

The mean scores of physical health subscales using SF-36 are presented in Table 1. As the table shows, the highest mean scores of physical health subscales, i.e. PF, BP, RP, and GH, were related to NVD. Kolmogorov-Smirnov test was used for checking the normality of the data (P=0.71). The results of one-way ANOVA showed no statistically significant difference among the three modes of delivery regarding the mean scores of physical health subscales.

**Table 1:** Mean scores of physical health subscales in the women with NVD, CS, and water birth using SF-36

**Quality**	**Modes of delivery**	**Mean±SD**	P value*
Physical Functioning	NVD	64.17±30.11	0.679
	Water birth	62.25±19.84	
	CS	59.51±24.54	
Role Physical	NVD	52.08±41.75	0.084
	Water birth	48.75±27.71	
	CS	36.25±30.98	
Bodily Pain	NVD	57.98±29.31	0.797
	Water birth	56.55±21.25	
	CS	57.65±24.25	
General Health	NVD	60.83±16.67	0.813
	Water birth	59.90±12.41	
	CS	59.01±13.76	
Total physical health score	NVD	57.83±20.32	0.677
	Water birth	57.61±11.33	
	CS	55.10±12.05	

*Test: One-way ANOVA

The mean scores of mental health subscales using SF-36 are shown in Table 2. As the table shows, NVD had the highest mean score in SF, water birth had the highest mean score in RE, and CS had the highest mean score in VT.

**Table 2:** The mean scores of mental health subscales in the women with NVD, CS, and water birth using SF-36

**Quality**	**Mode of delivery**	**Mean±SD**	P value*
Social Functioning	NVD	63.97±24.41	0.85
	Water birth	62.68±17.22	
	CS	61.53±19.33	
Role Emotional	NVD	53.87±44.75	0.001
	Water birth	57.48±29.37	
	CS	27.45±35.34	
Emotional well-being	NVD	64.20±15.8	0.299/797
	Water birth	66.50±15.2	
	CS	69.01±13.91	
Vitality	NVD	60.83±20.96	0.779
	Water birth	60.63±17.00	
	CS	63.00±9.32	
Total mental health score	NVD	60.17±18.76	0.247
	Water birth	61.41±11.16	
	CS	56.05±11.97	

*Test: One-way ANOVA

The results of one-way ANOVA revealed a significant difference among the three types of delivery concerning emotional problems (P<0.001) and Post Hoc test (Tukeys-b) showed a significant relationship between NVD and CS and also water birth and Cs (P<0.001). However, no statistically significant differences were found with respect to the other subscales.

The total QOL mean scores of the women who had had NVD, CS, and water birth were 58.92±19.51, 54.18±11.97, 59.38±11.21, respectively. Therefore, the women with water birth had the highest total QOL mean scores. However, the results of one-way ANOVA demonstrated no statistically significant differences among the three types of delivery in this regard (P=0.238). 

## Discussion

The results of the present study showed that the women with NVD had the highest mean scores in PF, BP, RP, and GH subscales; however, there were no statistically significant differences among the three groups. Similarly, a study compared NVD and CS and showed that the NVD group obtained higher scores in most physical health subscales (PF, RP, and BP). Yet, significant differences were observed only in PF and RP subscales.^21^ Moreover, another study also compared the women’s quality of life after CS and NVD at six weeks postpartum and indicated higher mean scores of physical health subscales in the NVD group.^16^ In the same line, a study in Iran indicated a significant difference between the CS and NVD groups concerning the mean scores of physical health subscales (58.27±13.39 vs. 66.78±13.41).^22^ However, a study in Sweden revealed that satisfaction with CS was higher than NVD because of the elective nature CS.^23^ Moreover, water birth had the second rank in physical health. Therefore, NVD and water birth showed the highest and second highest levels of physical health after delivery respectively, and should be considered as the best choices.

According to the findings of the present study, the NVD and water birth groups had the highest and second highest SF mean scores respectively. Abedian showed that the SF mean score was 44.3 in the CS group and 44.8 in the NVD group.^22^ Another study has confirmed this finding, reporting higher SF mean scores in the NVD group in comparison to the CS group.^18^ Since CS has negative psychological consequences for the mother, infant and family,^24^ the two other delivery methods are recommended to be used.

In the current study, the women in the water birth group had the highest mean score of RE subscale, indicating minimal emotional suffering in this group. A study conducted on 2000 women who had had water birth revealed noticeably high satisfaction with this type of delivery.^25^ The decrease in emotional suffering can be attributed to women’s high satisfaction with this new mode of delivery.

Our study findings showed that, although there were no statistically significant differences, the women with CS had the highest mean score of VT subscale; this is consistent with the results obtained by other studies.^18^^,^^26^ Conversely, Kalani found that there were not differences between NVD and CS in VT subscale.^27^ It seems that since tiredness and decreased energy are less common in case of CS, vitality is higher in CS.

The results also demonstrated that the CS group had the highest mean score in EW subscale (although there were no statistically significant differences). A study showed that fear of labor pain and being concerned about infant’s safety were the primary reasons for selecting CS.^28^ Hence, peace of mind and higher EW in the women undergoing CS can be the result of relief from these concerns. Saberi found that the EW mean score was higher among the women with CS.^21^ In contrast, a study indicated higher EW mean scores among the women in the NVD group.^18^^,^^26^

In the present study, nevertheless, there were no statistically significant differences; the women with water birth had the highest overall mental health mean scores (VT, SF, RE, and EW). This can be due to the lack of need for episiotomy or perineotomy in this mode of delivery.^1^^,^^29^ In other studies, NVD had a higher score than SC.^22^^,^^27^ There is no study on comparison of the effects of water birthin this area.

The highest mean score of total QOL was also related to the women who had undergone water birth (there was no statistically significant difference). In general, medical teams and pregnant women approve of water birth due to its comfort, more emotional support, safety of the mother and her infant, pain reduction, and decrease of medical intervention, such as oxytocin, episiotomy, and anesthesia.^4^^,^^11^ Moreover, decreased need for opiates, lower drug consumption, increased movement of the mother, as well as improved positioning in different stages of labor are other advantages of water birth.^30^^,^^31^

*Limitations*

The latent mental disorders of the participants were not estimated by the research team and we only trusted the participants’ answers. Limited number of studies in water birth for discussion is another limitation in this study. Another limitation was the small sample size, which was due to the limited number of women who had had waterbirth. 

## Conclusion

Women with water birth and NVD had the highest and second highest total QOL mean scores, respectively; women with NVD and water birth experienced better physical health than women who had had CS. In sum, governments can encourage women to choose water birth and NVD as the delivery modes with the highest postpartum QOL through health education. Moreover, it is recommended that families should be encouraged to select water birth and NVD in premarital educational courses, and the awareness of couples should be improved on the advantages and disadvantages of water birth, NVD and cesarean. 
